# Seventh European Workshop on Cannabinoid Research and IACM Eighth Conference on Cannabinoids in Medicine

**DOI:** 10.1089/can.2015.29001.jfc

**Published:** 2016-01-01

**Authors:** Joseph F. Cheer, Mauro Maccarrone, Daniele Piomelli

**Affiliations:** ^1^ Departments of Anatomy & Neurobiology and Psychiatry, University of Maryland School of Medicine, Baltimore, Maryland.; ^2^ Department of Medicine, Campus Bio-Medico University of Rome, and IRCCS Santa Lucia Foundation, Rome, Italy.; ^3^ Departments of Anatomy and Neurobiology, Pharmacology, and Biological Chemistry, University of California, Irvine, California.; ^4^ Istituto Italiano di Tecnologia, Genova, Italy.

**Keywords:** addiction, cannabis use disorder, endocannabinoid, medicinal cannabis, pain, stress

## Abstract

The joint 7th European Workshop on Cannabinoid Research and IACM 8th Conference on Cannabinoids in Medicine was held in the beach town of Sestri Levante, Italy, on September 17–19, 2015. In this beautiful setting, world-leading investigators in the field of (endo)cannabinoid research presented exciting new data spanning a broad array of preclinical and clinical topics—from cellular electrophysiology to drug discovery and from potential indications for the therapeutic use of cannabis to treatments for cannabis use disorder. The sheer breadth of interests represented at the meeting generated a lively debate, which filled not only the question–answer time after the talks, but also the coffee and lunch breaks in the sunny courtyard of the ancient monastery where the conference was held. The (endo)cannabinoid research field is varied and expanding, and many participants voiced their support for having a forum where diverse (and sometimes contrasting) ideas can be exchanged, and new collaborations can be started. The active participation of both basic and clinical scientists was unanimously recognized as a major strength of this important scientific event.

## Introduction

In late summer of 2015, world-leading preclinical and clinical investigators in the field of cannabinoid research converged upon the Bay of Silence, one of the most iconic and breathtaking landmarks of the Liguria region in northern Italy. A beach surrounded by beautiful buildings and a clear blue sea were conducive to an outstanding exchange of ideas and divulgation of new and exciting findings in this timely field of scientific inquiry. The three-day meeting organized by Drs. Franjo Grotenhermen, Giovanni Marsicano, Miriam Melis, and Daniele Piomelli was divided into eight sessions and featured two plenary lectures, the content of which is summarized below.

## Plenary Lecture I (Day 1)

Dr. D. Piomelli (University of California, Irvine, and Italian Institute of Technology, Genoa) kicked off the meeting with a quick overview of the history of cannabinoid research. He shared a personal vision of the status of the field and pointed out that the discovery of new cannabinoid-based medicines can only start from a deeper understanding of the endocannabinoid signaling system. He exemplified this idea with an example taken from the work in his lab, which has recently demonstrated an unexpected role for the endocannabinoid anandamide in the regulation of social reward.

## Neural Circuits (Session 1, Day 1)

The conference continued with a series of modern neuroscience talks by established and new investigators, all aimed at identifying new physiological functions and anatomical substrates whereby (endo)cannabinoid compounds may exert their effects within the central nervous system. Dr. J. Bains (University of Calgary, Canada) reported that endocannabinoid-mediated plasticity in the hypothalamus may be driven by relative salience (i.e., stress). Next, Dr. R. Tonini (Italian Institute of Technology, Genoa) provided new data suggesting that activation of endocannabinoid-dependent changes in dorsal striatal plasticity has the potential not only to change striatal output function but also to restore motor function.

An excellent anatomical perspective provided by Drs. Dudok and I. Katona (Hungarian Academy of Sciences, Budapest) followed this exciting presentation. They introduced a correlative approach based on STORM super-resolution and confocal microscopy that allows the nanoscale molecular profiling of axon terminals of individually characterized hippocampal neurons. By using this new methodology, they have identified cell type-specific principles in the nanoscale configuration of (endo)cannabinoid signaling between glutamatergic pyramidal cells versus perisomatically and dendritically targeting GABAergic interneurons. Moreover, this allows for the visualization of the robust downregulation and long-lasting reorganization of type 1 cannabinoid (CB_1_) receptors after chronic Δ^9^-THC treatment in specific axon terminals.

Two presentations focused on feeding control followed the anatomical perspective and closed the morning session. In the first one, Dr. D. Cota (INSERM, Bordeaux, France) examined intracellular mechanisms in the hypothalamic regulation of energy balance, and how this relates to the melanocortin system. Dr. T. Horvath (Yale University, USA) spoke about how pro-opiomelanocortin cells in the arcuate nucleus of the hypothalamus promote gradual onset of satiety, and how this system can explain, in part, the promotion of feeding by exogenous cannabinoids such as Δ^9^-THC.

The afternoon session began with a series of talks on miscellaneous topics, the first of which was delivered by Dr. J. Gertsch (University of Bern, Switzerland). He summarized a recent discovery by his team that the peptide pepcan-12, a prototypical cannabinoid allosteric modulator, protects tissues from overstimulation of CB_1_ receptors and understimulation of CB_2_ receptors. Dr. M. Bagelaar (University of Leyden, The Netherlands) discussed the identification, via activity-based chemoproteomics assays, of a novel reversible dual diacylglycerol lipase (DAGL-α/DAGL-β) inhibitor, the α-ketoheterocycle LEI105, in which broad-spectrum and tailor-made activity-based probes are combined to report on inhibitor activity and selectivity. Targeted lipidomics further revealed that LEI105 concentration dependently reduced 2-arachidonoylgylcerol (2-AG) levels, but not anandamide levels, in Neuro2A cells. Using this approach, Dr. Bagelaar showed that CB_1_ receptor-mediated short-term synaptic plasticity in a mouse hippocampal slice model was reduced by LEI105.

Next, Dr. Z. Lenkei (ESPCI-ParisTech, France) provided evidence that long-term decreases in neurotransmitter release controlled by CB_1_ receptor activation depend upon Rho-associated protein kinase-mediated actomyosin contraction. This exciting first day ended with three presentations from Dr. G. Marsicano's team (INSERM, Bordeaux, France): Dr. Desprez presented new results pointing to a role for mitochondrial CB_1_ receptors in the regulation of memory processes; Dr. C. Muguruza showed how the endocannabinoid system controls motivation for voluntary physical exercise in mice; and Dr. A. Busquets-Garcia proposed that central and peripheral cannabinoid mechanisms regulate the impact of stress on memory formation.

## Neurons, Glia, and Brain Dysfunction (Session 2, Day 2)

Dr. T. Nevian (University of Bern, Switzerland) opened the second day of the meeting with the proposal that endocannabinoids may underlie certain effects on plasticity at cortical synapses, and that they may act by recruiting astrocyte signaling. Along similar lines, Dr. A. Araque (University of Minnesota) provided evidence that endocannabinoids may engage astrocytes to produce various forms of synaptic plasticity in the brain. Next, Dr. S. Marinelli (European Brain Research Institute, Rome, Italy) showed provocative data suggesting that CB_2_ receptors may regulate cortical inhibition. Session 2 ended with a presentation by Dr. O. Manzoni (INSERM, Marseille, France), who described how imbalances in brain polyunsaturated fatty acid levels have important implications on cortical neurotransmission and cognitive function. He also reported data suggesting that pharmacological modulation of endocannabinoids has the potential to rectify behavioral and neural deficiencies caused by deficits in polyunsaturated fatty acids.

## Pain (Session 3, Day 2)

Dr. D. Finn (National University of Ireland, Galway) presented the results of recent studies aimed at elucidating the role of the endocannabinoid system in discrete brain regions in the bidirectional modulation of pain by negative affective state. The data confirm that the endocannabinoid system plays a key role in mediating fear-induced analgesia by acting in circuits located in the dorsolateral periaqueductal gray (PAG), basolateral amygdala, ventral hippocampus, and medial prefrontal cortex. Dr. Finn also reported that anxiety/depression-related hyperalgesia in the stress-hypersensitive Wistar-Kyoto rat strain is associated with deficits in endocannabinoid signaling in the PAG-rostroventromedial medulla circuitry. This presentation was followed by Dr. V. Chapman's (University of Nottingham, UK), who provided an update on (endo)cannabinoid actions in several types of pain conditions.

Several talks by young investigators followed this session. Dr. A. Ruiz-Calvo (Complutense Unviersity, Madrid) summarized her research into the neuroprotective role of CB_1_ receptors present on striatal astrocytes. Because astrocytes are crucial for neuronal physiology and their deregulation occurs in many neurodegenerative diseases, she posited that this particular cell type is a relevant target to apply cannabinoid-based therapies. Next, Dr. F. Papaleo (Italian Institute of Technology, Genoa) showed, using genetically modified mice that carry the COMT-Val human variant (i.e., COMT-Val transgenic mice), that a unique COMT Val–cannabis interaction may contribute to the development of schizophrenia-relevant phenotypes with a crucial timing component of adolescence as a vulnerability risk period. The morning session ended with a presentation by Dr. C. Bosch-Bouju (Bordeaux University, France) on the relationship between endocannabinoid synaptic plasticity and the development of anxiety in mice subjected to chronic social defeat stress.

## Immune and Metabolic Control (Session 4, Day 2)

The afternoon session began with Dr. B. Lutz's (Mainz University, Germany) description of an anatomical and physiological framework for the role of CB_1_ receptors present in cortical glutamatergic neurons, central serotonergic neurons, adrenergic or noradrenergic cells, and adipocytes to provide an overview of the multifaced roles of CB_1_ receptor activation in the control of energy balance.

Dr. V. Chiurchiù (Campus Bio-Medico University of Rome, Italy) then went on to discuss how the two main endocannabinoid substances present in the brain—anandamide and 2-AG—may exert distinctive immunomodulatory effects on specific cell lineages involved in innate and adaptive immunity. In particular, anandamide may be able to suppress T-helper (TH)-1 and TH-17 responses either directly or through inhibition of dendritic cells, to impact the balance between M1 and M2 macrophages and dampen the inflammatory influences of specific subsets of dendritic cells. On the other hand, 2-AG mainly exerts activatory functions, notably on macrophages and regulatory T cells. These cell-specific immunomodulatory effects are significantly altered in several chronic inflammatory diseases, including neurodegenerative diseases and metabolic disorders, and are due to a different reorganization of CB_2_ receptors and anandamide-degrading enzyme fatty acid amide hydrolase in immune cells.

The session ended with Dr. S. Lotersztajn's (Paris Diderot University, France) presentation of the role of endocannabinoid signaling in the liver. She specifically emphasized how inhibitors of the 2-AG-degrading enzyme monoacylglycerol lipase are potent anti-inflammatory and antifibrogenic molecules in the liver.

## Stress, Addiction, and Memory (Session 5, Day 2)

Dr. M. Pistis (University of Cagliari) began the session by presenting data suggesting that activation of nuclear peroxisome-proliferator-activated receptor-α (PPAR-α) might exert antidepressant-like properties. Fenofibrate, a PPAR-α agonist currently prescribed as lipid-lowering medication, was chronically administered in a rat model of stress-induced depression and prevented the development of depressive-like symptoms in stressed animals. Importantly, these findings were accompanied by an enhancement of dopamine and serotonin neuronal activity. After this presentation, Dr. D. Parolaro (University of Insubria, Italy) spoke about the enduring effects of cannabinoids when exposure occurs in adolescence, with a special emphasis on epigenetic mechanisms. Her data support the notion that adolescent cannabinoid exposure disrupts remodeling of cortical circuits, thus leading to behavioral abnormalities. Changes in both histone modification and gene expression were more pronounced after adolescent treatment compared to adults, further supporting higher adolescent vulnerability to the enduring effects of Δ^9^-THC.

Dr. A. Ozaita (Pompeu-Fabra University, Spain) then shared new data showing that the endocannabinoid system can normalize mTOR signaling in the brain in a rodent model of intellectual disability disorders, suggesting that it is a suitable target for the design of therapies that improve cognitive performance in different intellectual disability disorders, independently of their genetic etiology. The session was closed by Dr. P.V. Piazza (INSERM, Bordeaux, France), who reviewed the roles of pregnenolone as a signaling-specific cannabinoid receptor modulator, and disclosed a novel pregnenolone derivative that might find use in the treatment of cannabis use disorders.

## Plenary Lecture II (Day 3)

For this second plenary of the meeting, Dr. V. Di Marzo shed new light on a previously understudied topic, namely, the role of endocannabinoids in skeletal muscle cell differentiation. His group recently showed that activation of CB_1_ receptors by 2-AG prevents myotube formation in a manner reversed by CB_1_ knockdown or pharmacological blockade, which, *per se*, stimulates differentiation, similar to the inhibition of 2-AG biosynthesis. He went on to present data suggesting that CB_1_ receptors also play a role in myotube formation from skeletal muscle satellite cells, where the receptor is upregulated by PAX7 and dysregulated during muscle dystrophy as in Dmd^*mdx*^ mice. He concluded that CB_1_ receptor manipulations could theoretically ameliorate muscular function in conditions such as Duchenne's muscular dystrophy.

## Cannabis-Based Medicines: Some Potential Applications (Session 6, Day 3)

Dr. R. Pertwee (University of Edinburgh, UK) started this set of translational talks by providing functional evidence that phytocannabinoids enhance 5HT1A receptor activity and raised the interesting possibility that these agents could potentially induce 5HT1A receptor-mediated reductions in signs of schizophrenia-like behavior in preclinical models. This was followed by Dr. E. Russo's (Phytecs, USA) historical overview of the use of the marijuana plant for a number of conditions. He also presented new findings highlighting three new areas of therapeutic interest—tetanus, tinnitus, and burn treatment.

For a cancer perspective, Dr. G. Velasco (Complutense University, Spain) discussed the conditions under which cannabinoids can be used therapeutically as anticancer agents, based upon the ability of these compounds to stimulate autophagy-induced cancer cell death. Dr. M. Maccarrone (Campus Bio-Medico University of Rome, Italy) delivered the morning session's concluding presentation. He showed evidence that a full and functional endocannabinoid system in the skin and in its adnexal structures (e.g., hair follicle, sebaceous, and sweat glands) exists. He also showed that (endo)cannabinoids regulate skin physiology, including the regulation of proliferation, differentiation, and survival of skin cells, of sebum production and melanogenesis, as well as of immune competence and/or tolerance of keratinocytes. Moreover, cutaneous CB_1_ receptors were shown to limit secretion of proinflammatory chemokines, suggesting that they might regulate T-cell-dependent inflammatory diseases of the skin as well (e.g., psoriasis, contact dermatitis, and atopic dermatitis). Therefore, new clues on the relevance of endocannabinoid signaling at the skin–immune cell interface were discussed along with new data on the impact of CB_1_-mediated mTOR inhibition on cytokine profile of inflamed human keratinocytes.

The afternoon talks initially documented the actions of VSN16R, a cannabinoid-based drug for the treatment of spasticity (Dr. D. Baker, Queen Mary University, UK). Next, Dr. M. Sexton (Bastyr University, USA) shared data on an international survey of medical cannabis use. In the largest survey of its type, with 1331 participants, an average symptom improvement of 3.96 on a scale of −5 (worsening) to +5 (improving) was reported across conditions. The majority of participants were male (55%). The most common symptoms for which participants used cannabis were pain, anxiety, depression, headache/migraine, and arthritis. Inhalation was the preferred route of administration (3–7 g of cannabis per week). Quality-of-life measures by PROMIS global method were on par with the general population for mental health and one standard deviation below for physical health, expected for medical users. Finally, young investigator Dr. A. Olah (University of Debrecen, Hungary) showed findings that fatty acid amide hydrolase inhibitors (URB597 and JP104) exert complex anti-acne effects, most probably via the downregulation of c-Myc and nuclear receptor-interacting protein 1, and activation of PPARs. He concluded by inviting clinical studies to exploit their potency as promising, novel class anti-acne agents.

## Pharmaceutical Perspectives of Cannabinoid-Based Medicines (Session 7, Day 3)

Dr. J. Brodie (GW Pharmaceuticals, U.K.) compared a combined cannabis extracts approved as a single medicine (Sativex^®^) for spasticity in multiple sclerosis, with pure cannabidiol (Epidiolex^®^), a multitarget cannabinoid that has recently emerging as a treatment for pediatric drug-resistant epilepsy with recently reported clinical results in ulcerative colitis. He underscored the concept of polypharmacology and described a new empirical model, the “therapeutic handshake,” to predict efficacy and safety of compound combinations of either natural or synthetic origin. Dr. T. Erkelens (Bedrocan, The Netherlands) provided an update on Bedrocan, an international producer of pharmaceutical-grade cannabis. Topics covered included standardized cultivation (cultivation, processing, packaging), quality control (QC lab), chemical profiling (cannabinoids and terpenes), administration forms (oil, vaporizing, tea), botanical research (*Cannabis sativa* vs. *Cannabis indica* varieties), social studies (patient preferences), and clinical research (chronic pain).

## Clinical and Patient Perspective of Cannabinoid-Based Medicines (Session 8, Day 3)

The session started with a retrospective description of clinical nabilone use in the United Kingdom by Dr. W. Notcutt (Great Yarmouth, UK), who showed that the vast majority of nabilone use was in unlicensed indications. Although most patients who were prescribed nabilone for multiple sclerosis had experienced symptoms for many years and had been prescribed a number of other medications, over half derived benefit and adverse effects were no different from other CNS-active medications. Next, Dr. M. Ware (McGill University, Canada) showed that medical education of health professionals regarding medical cannabis in Canada remains a significant challenge, met in part by several online accredited programs and conferences. This was contrasted by Dr. F. Grotenhermen's (Nova Institute Huerth/Rhineland, Germany) seminar on the use of medical cannabis in Germany. In particular, he noted that there is a severe undersupply of the German population with cannabis-based medicines compared to Canada, the Netherlands, and Israel.

## Concluding Remarks

Participants concurred that a forum for exchanging ideas and fostering new collaborations in the ever-growing field of (endo)cannabinoid signaling such as this one was much needed. The merging of basic scientists and clinicians was unanimously recognized as a key strength for gaining a better understanding of the potential therapeutic exploitation of (endo)cannabinoids in several human diseases. Even though evaluation forms for the meeting were not provided—a weakness that should be corrected in the future—the general atmosphere was one of interest and satisfaction. The high-quality and varied perspectives offered by the speakers—as well as the richness of the discussion—underscore the maturity of the (endo)cannabinoid research field and its readiness to take on the medical and societal challenges lying ahead.

## Abbreviations Used

2-AG: 2-arachidonoylgylcerol
CB1: type 1 cannabinoid
PAG: periaqueductal gray
PPAR-α: peroxisome-proliferator-activated receptor-α
TH: T-helper

